# Major Differences in Lymphocyte Subpopulations Between Cerebrospinal Fluid and Peripheral Blood in Non-Hodgkin Lymphoma Without Leptomeningeal Involvement: Flow Cytometry Evidence of a Cerebral Lymphatic System

**DOI:** 10.3389/fonc.2021.685786

**Published:** 2021-06-03

**Authors:** Iole Cordone, Serena Masi, Diana Giannarelli, Alessia Pasquale, Laura Conti, Stefano Telera, Andrea Pace, Elena Papa, Mirella Marino, Paolo de Fabritiis, Andrea Mengarelli

**Affiliations:** ^1^ Department of Research, Advanced Diagnostics and Technological Innovation, IRCCS Regina Elena National Cancer Institute, Rome, Italy; ^2^ Department of Research and Clinical Oncology, IRCCS Regina Elena National Cancer Institute, Rome, Italy; ^3^ Hematology, S Eugenio Hospital, ASL Roma2, Tor Vergata University, Rome, Italy

**Keywords:** cerebrospinal fluid, lymphocytes, flow cytometry, NHL, cerebral lymphatic system

## Abstract

Cerebrospinal fluid (CSF) flow cytometry has a crucial role in the diagnosis of leptomeningeal disease in onco-hematology. This report describes the flow cytometry characterization of 138 CSF samples from patients affected by non-Hodgkin lymphoma, negative for disease infiltration. The aim was to focus on the CSF non-neoplastic population, to compare the cellular composition of the CSF with paired peripheral blood samples and to document the feasibility of flow cytometry in hypocellular samples. Despite the extremely low cell count (1 cell/µl, range 1.0–35) the study was successfully conducted in 95% of the samples. T lymphocytes were the most abundant subset in CSF (77%; range 20–100%) with a predominance of CD4-positive over CD8-positive T cells (CD4/CD8 ratio = 2) together with a minority of monocytes (15%; range 0–70%). No B cells were identified in 90% of samples. Of relevance, a normal, non-clonal B-cell population was documented in 5/7 (71%) patients with primary central nervous system lymphoma at diagnosis (*p*<0.0001), suggesting a possible involvement of blood-brain barrier cell permeability in the pathogenesis of cerebral B-cell lymphomas. The highly significant differences between CSF and paired peripheral blood lymphoid phenotype (*p*<0.0001) confirms the existence of an active mechanism of lymphoid migration through the meninges.

## Introduction

Neoplastic meningitis is a dramatic complication in cancer patients and the diagnosis of leptomeningeal metastasis represents one of the greatest challenges in neuro-oncology. Cerebrospinal fluid (CSF) analysis has a key role in routine clinical practice however conventional cytology, the gold standard for cell type identification, has considerable limitations regarding sensitivity and specificity, with a reported false-negative rate of up to 40% (1, 2).

In recent years, several studies have demonstrated that CSF flow cytometry is superior to conventional cytology for detection of CNS involvement in non-Hodgkin lymphomas, acute leukemia and multiple myeloma (3–11). Thereafter, flow cytometry is recognized among the basic elements for the diagnosis of leptomeningeal metastasis in hematologic cancers (12, 13), although the low cell count of CSF samples, combined with the rapidly declining leukocyte viability, makes CSF flow cytometry challenging (14). More recently, flow cytometry application and efficiency in diagnosis of solid tumors leptomeningeal metastasis is gaining more evidence (15–18).

However, cancer cells represent only a proportion, often a minority, of the CSF population in neoplastic meningitis. A significant presence of lymphocytes has been documented, together with floating malignant cells, in CSF samples from patients with non-Hodgkin lymphomas and breast cancer leptomeningeal metastasis (18, 19); an active mechanism of reactive CD8 T-lymphocyte migration has been observed in primary central nervous system lymphomas (PCNSL) of B-cell type (20, 21). These findings suggest an active role of the central nervous system (CNS) lymphatic system in both lymphoid and tumor cells migration into and out of the meninges.

Focusing on non-neoplastic populations, we report here the immunophenotype of the CSF leukocytes of patients affected by non-Hodgkin lymphomas without leptomeningeal involvement. The aim was to document the feasibility of flow cytometry in normal, thereafter, extremely hypocellular samples, to document the immunophenotype of CSF non-neoplastic population in non-Hodgkin lymphoma, to compare the cellular composition of the CSF with paired peripheral blood samples. Moreover, a possible correlation between the CSF lymphocyte subpopulations and diagnosis was evaluated.

## Materials and Methods

### Patients

From March 2010 to December 2015 a cohort of 138 samples with non-Hodgkin lymphoma who underwent diagnostic lumbar puncture according to the routine clinical practice entered the study (22). All PCNSL cases diagnosed until December 2019 were also included. Lymphomas were classified according to the World Health Organization (WHO) classification (23).

All CSF samples were analyzed by cytology and flow cytometry and had no evidence of infiltration. Patients with a positive diagnostic lumbar puncture due to CSF infiltration by pathological cells were excluded from this analysis. The Central Ethical Committee IRCCS Lazio, Section I.F.O. approved this retrospective study. Protocol n° 0009524, July 27^th^ 2020.

### CSF Collection and Cell Count

CSF was collected in a tube without any transport medium or anticoagulant and processed within 1 to 3 h to minimize cell loss. To avoid peripheral blood contamination, the first 0.2 to 0.4 ml (four to eight drops) of CSF were discarded before sample collection. A standard cell count was performed using the Turk reagent and a Nageotte chamber. CSF was spun at 1,400 rpm for 5 min, the supernatant fluid was discarded and the cell pellet was suspended in 500 µl of phosphate buffered saline (PBS): 100 µl of cell suspension was used for cytomorphology and 100 µl/tube for the flow cytometric study.

### CSF Morphological Evaluation

Cytospins were prepared using a Shandon CytoSpin cytocentrifuge. Morphological examination was performed by expert cytopathologists using May–Grünwald–Giemsa staining. All cases were morphologically negative for CNS localization.

### CSF Flow Cytometry Assay and Analysis

CSF samples were processed and stained using a 6-color monoclonal antibodies panel, 5 µl of each, and the “Duo-lyse” program of the Becton Dickinson Bioscience (BDB) Lyse-Wash-Assistant according to the following combinations: tube 1) CD3Fitc (BD Biosciences Cat# 345763, RRID : AB_2811220), CD56Pe (BD Biosciences Cat# 345812, RRID : AB_2629216), CD45PerCP-Cy5.5 (BD Biosciences Cat# 332784, RRID : AB_2868632), CD4PE-Cy7 (BD Biosciences Cat# 348809, RRID : AB_2783789), CD19APC (BD Biosciences Cat# 345791, RRID : AB_2868817) and CD8APC-Cy7 (BD Biosciences Cat# 348813, RRID : AB_2868857); tube 2) CD5Fitc (BD Biosciences Cat# 345781, RRID : AB_2868807), CD10Pe (BD Biosciences Cat# 332776, RRID : AB_2868625), CD45PerCP-Cy5.5, CD2PE-Cy7 (BD Biosciences Cat# 335821, RRID : AB_2868684), CD79bAPC (BD Biosciences Cat# 335834, RRID : AB_2868695) and CD20APC-Cy7 (BD Biosciences Cat# 335829, RRID : AB_2868690); tube 3) anti-Lambda Fitc (BD Biosciences Cat# 347247, RRID : AB_2868845, anti-Kappa Pe (BD Biosciences Cat# 347246, RRID : AB_2868844), CD45PerCP-Cy5.5, CD34PE-Cy7 (BD Biosciences Cat# 348811, RRID : AB_2868855), CD22APC (BD Biosciences Cat# 333145, RRID : AB_2868646) and CD14APC-Cy7 (BD Biosciences Cat# 333951, RRID : AB_2868679). All antibodies were from BDB. Prior to sample acquisition, a flow cell cleaning with FACS flow (for 1 to 2 min run) was performed to avoid any event carry over. The whole volume of sample was acquired and analyzed using the FACSCanto II 2L flow cytometer and the FACSDiva software Version 6.1.3 (BDB). Single-stained cellular controls, BD FACS™ 7-color setup beads and BD FACSDiva CS&T IVD Beads have been used to adjust detector voltage, to set fluorescence compensation and to monitor instrument performance.

Data are presented as the percentage of positive cells evaluated on the CD45-positive population. Lymphocytes were identified by CD45-strong/side scatter (SSC)-low. The CD4 and CD8 subsets were evaluated as a percentage of CD3-positive T lymphocytes. Monocytes were identified using the CD4-weak or CD14 staining. Neutrophils as CD45/SSC-high. Surface immunoglobulins (Ig) kappa and lambda light chain expression was evaluated on CD22-positive B cells. In agreement with the recommendations for the analysis of rare events, a cluster of 10 events was considered to define a positive result and identify a leukocyte subpopulation (10). Disease infiltration of the CSF was excluded, being negative for the lymphoma-associated phenotype identified by histopathology. We defined as CSF negative all sample negative by cytology and flow cytometry.

The peripheral blood lymphocyte characterization, using the CD3 CD56 CD45 CD4 CD19 and CD8 combination (tube n°1), was conducted in 104 paired cases.

### Statistical Analysis

Qualitative items were reported as absolute and percentage counts, while quantitative variables were summarized using mean and standard deviation, median and range. The difference in distribution between CSF and PB lymphoid subpopulations was assessed by Wilcoxon rank-sum test. Association between variables was evaluated with the Spearman’s ρ coefficient. The test was two-sided with a *p*-value of <0.05 indicating a statistically significant difference. All statistical analyses were performed using SPSS (version 21.0).

## Results

### Patients

A cohort of 138 samples from 127 non-Hodgkin lymphoma patients, all negative for CNS disease involvement, entered the study (**Table 1**). Eighty-three patients (65%) were male and median age was 60 years (range 18–84). The lumbar puncture for analysis was performed at diagnosis (n=108), at follow up (n=11), at relapse (n=12), or with progressive disease (n=7).

**Table 1:** Diagnostic distribution of 127 non-Hodgkin lymphoma patients who underwent diagnostic lumbar puncture according to the routine clinical practice and were negative for leptomeningeal involvement.

Diagnosis	Number of cases
DLBCL	90
MCL	14
PCNSL	11
FL	5
Anaplastic large cell lymphoma	2
Peripheral T-NHL	2
Burkitt lymphoma	1
LBL	1
T-cell rich B-cell lymphoma	1

DLBCL, diffuse large B-cell lymphoma; MCL, mantle cell lymphoma; PCNSL, primary central nervous system lymphoma; FL, follicular lymphoma; LBL, lymphoblastic lymphoma.

The study focuses on 107 cases; 24 cases (17%) were not included in analysis due to peripheral blood contamination of the CSF documented by the identification of red blood cells in the cytospin assessed morphologically as well as a population of CD45/SSC high (46%; range 30–89%) positive neutrophils. In seven cases the flow cytometry analysis was not evaluable due to the absence of clustered events.

### Immunophenotype of CSF Sample

A median volume of 4.0 ml (range 2.0–12.0) of CSF was available for flow cytometry analysis. The CSF cell count was extremely low (1 cell/µl, range 1.0–35); in 9 cases (8%) the CSF cell count was higher than the normal reference value of 4 cell/µl, with a median value of 22 cells/µl (5.0–35). Despite the low absolute cell number, flow cytometry characterization was successfully conducted in 95% of cases (131/138).

**Figure 1:**
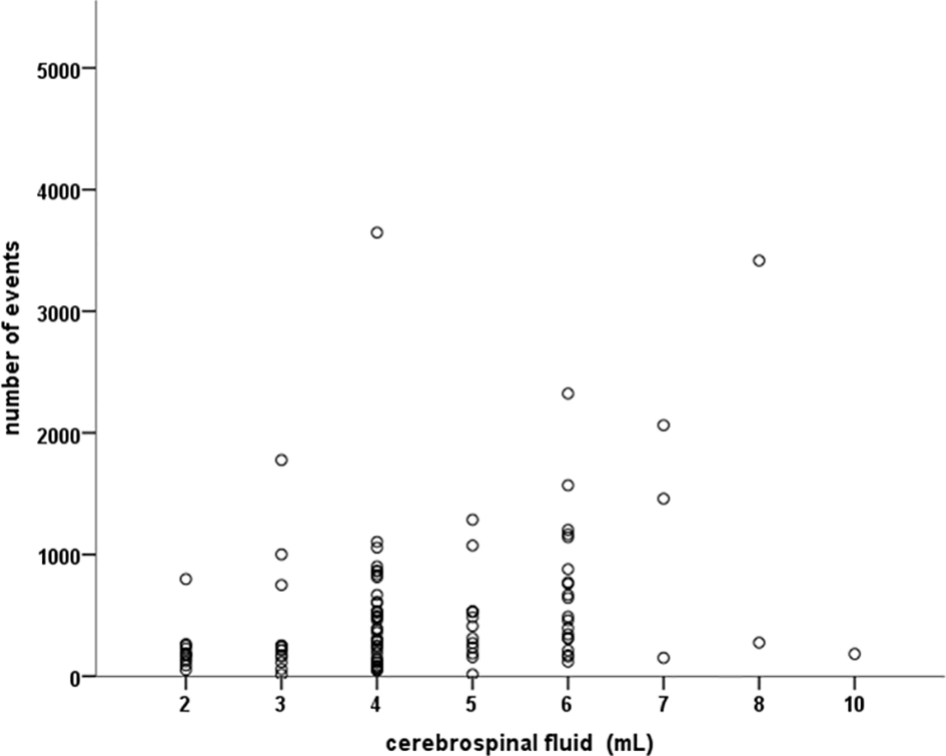
Positive correlation between the ml of cerebrospinal fluid and the number of cells (events) analyzed by flow cytometry (p 0.36; P < 0.001).

Gating on the CD45-positive population in combination with the side scatter, a median of 384 (range 49–23649; mean 1518 ± 3772) events were acquired and analyzed. A positive correlation was found between the volume (ml) of CSF and the number of events analyzed by flow cytometry (Δ 0.36; *p*<0.001) (**Figure 1**).

The CSF population was represented by lymphocytes (77%; range 20–100%) together with a minor population of monocytes (CD4-weak or CD14-positive 15%; range 0–70%).

The CSF lymphoid population was represented by CD2 CD3 CD5-positive T cells (94%; range 62–100%) with a prevalence of CD4-positive lymphocytes (CD4/CD8 ratio = 2). A minority of CD56-positive cells were also documented (5%; range 0–47%). No B cells (< 10 clustered events) were identified in 90% of cases (**Table 2**).

**Table 2:** Cerebrospinal fluid (CSF) and corresponding peripheral blood (PB) lymphoid immunophenotype comparison in 104 non-Hodgkin lymphoma patients negative for leptomeningeal involvement.

%	CSF median	CSF mean	PB median	PB mean	*p* CSF *vs* PB
Lymphocytes	78 (20–100)	71.1 ± 19.8	19 (2–76)	20.9 ± 12.0	<0.0001
CD19+	0 (0–22)	0.4 ± 2.5	4.5 (0–29)	6.5 + 6.6	<0.0001
CD3+	94 (62–100)	92.3 ± 6.9	72 (27–98)	71.8 + 12.7	<0.0001
CD3+/CD4+	65 (3–95)	62.6 ± 16.0	55 (2–86)	53.9 + 14.8	<0.0001
CD3+/CD8+	32 (4–81)	33.5 ± 14.9	38 (15–96)	40.1 + 14.7	<0.0001
CD4/CD8 *ratio*	2 (0.04–23.7)	–	1.4 (0.02–5.7)	–	<0.0001
CD56+	5 (0–47)	7.8 ± 8.9	21(1–61)	23.1 ± 12.8	<0.0001

Values are expresses as a percentage of CD45-positive lymphocytes. Wilcoxon rank-sum test was conducted to evaluate the different distribution between CSF and PB lymphoid subpopulations.

**Figure 2:**
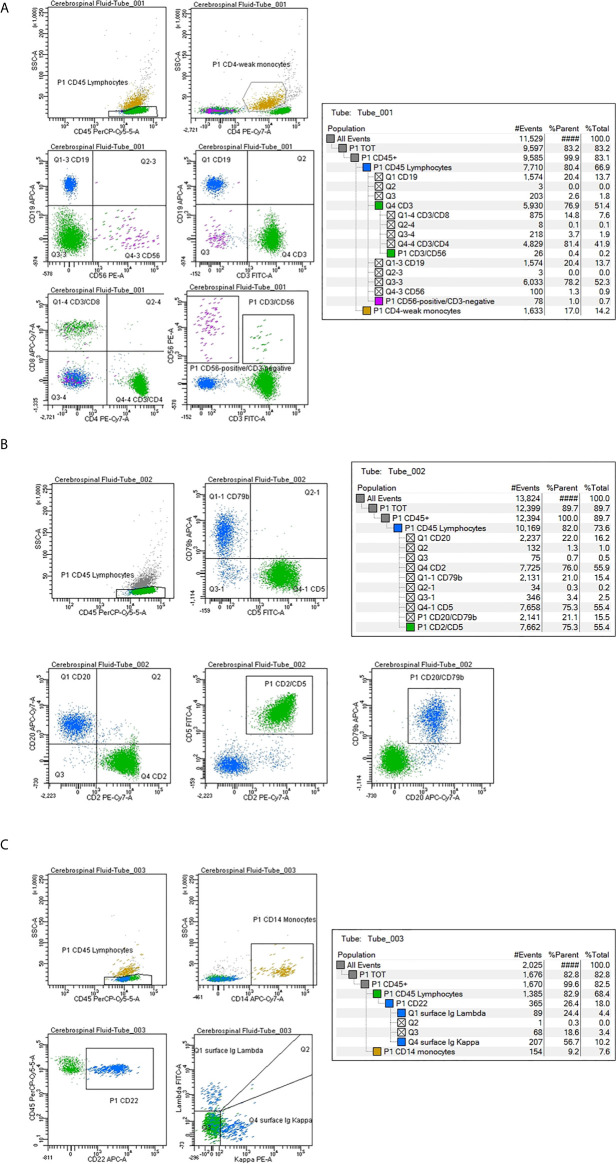
Cerebrospinal fluid (CSF) flow cytometry characterization in a case of diffuse large B-cell lymphoma negative for disease infiltration. Cell count: 24 cells/µl. The CSF lymphocyte immunophenotype is reported as percentage of positive cells within the lymphoid population, identified as CD45-strong/low SSC. **(A)** Tube number 1: Green color has been utilized to mark CD3 CD4 CD8-positive T lymphocytes; purple for CD56-positive cells, blue for CD19-positive B lymphocytes and dark yellow for CD4-weak monocytes. **(B)** Tube number 2: Green color has been utilized to mark CD2 CD5-positive T lymphocytes; blue for CD79b CD20-positive B cells. **(C)** Tube number 3: Blue color has been utilized to mark CD22-positive B lymphocytes. The Ig light chain expression shows a normal kappa/lambda ratio. Dark yellow marks CD14-positive monocytes.

In 11 patients a subpopulation of CD19 CD20 CD22 CD79b-positive B lymphocytes (4%; range 1–22%), with a normal/balanced Ig kappa/lambda ratio evaluated on the CD22-positive population, was identified: 5 PCNSL, 5 diffuse large B-cell lymphoma (DLBCL), 1 follicular lymphoma (**Table 3**) (**Figures 2A–C**). All 11 patients were at diagnosis. Non-clonal B lymphocytes were documented in 45.5% (5/11) of PCNSL, in 5.5% (5/90) DLBCL and 1/5 FL cases. Seven PCNSL were at diagnosis and 4 in disease progression; normal B cells were present in 5/7 (71%) PCNSL at diagnosis, identifying a significant correlation between a non-clonal B-cell subpopulation in the CSF and a diagnosis of cerebral B-cell lymphoma (*p*<0.0001).

**Table 3:** Analysis of the cerebrospinal fluid (CSF) and corresponding peripheral blood (PB) lymphocytes of 11/107 non-Hodgkin lymphoma patients, negative for leptomeningeal involvement, where a subpopulation of B cells has been identified at diagnosis by flow cytometry of the CSF sample.

	Diagnosis Case number	PCNSL 1	PCNSL 2	PCNSL 3	PCNSL 4	PCNSL 5	DLBCL 1	DLBCL 2	DLBCL 3	DLBCL 4	DLBCL 5	FL
**CSF**	**ml of sample**	5	5	5	12	4	4	3.5	7.5	4	7	5.5
	**Cell count/ µl**	1	16	1	3	35	2	nd	1	5	24	30
	**Number of events**	412	8583	1286	8285	21000	521	1105	6049	3647	23649	14072
	**Lymphocyte %**	70	80	77	85	95	80	75	91	90	82	92
	**Monocytes %**	15	16	17	11	5	19	14	6	8	17	5
	**CD2%**	86	98	95	95	93	94	95	97	98	76	89
	**CD3%**	82	91	93	91	90	94	91	97	96	77	87
	**CD5%**	73	90	95	89	87	90	90	95	96	75	86
	**CD56%**	3	9	2	4	5	7	7	3	3	1	1
	**CD3/CD4%**	79	62	47	78	75	66	69	73	79	81	65
	**CD3/CD8%**	21	32	48	21	24	32	32	25	20	15	31
	**T4/T8 ratio**	3.7	1.9	1	3.7	3.1	2.1	2.1	2.9	3.9	5.4	2.1
	**CD19%**	**8**	**1**	**5**	**3**	**6**	**3**	**4**	**1**	**2**	**20**	**12**
	**CD20%**	**9**	**1**	**4**	**4**	**6**	**4**	**2**	**1**	**3**	**22**	**11**
	**CD79b %**	**9**	**1**	**3**	**3**	**5**	**4**	**2**	**1**	**3**	**21**	**11**
**PB**	**Lymphocyte count/ µl**	2300	1120	1350	730	nd	1500	1700	2100	1780	2100	900
	**Lymphocyte %**	16	11	13	8	nd	20	30	25	37	14	11
	**CD3%**	71	68	72	88	nd	70	75	64	77	57	64
	**CD56%**	7	18	13	9	nd	21	11	28	7	55	16
	**CD3/CD4%**	62	64	40	62	nd	64	54	47	65	38	66
	**CD3/CD8%**	36	33	55	34	nd	31	45	44	32	60	33
	**T4/T8 ratio**	1.7	1.9	0.7	1.8	nd	2.0	1.2	1.1	2.0	0.6	2
	**CD19%**	22	13	17	9	nd	12	11	9	11	2	21

PCNSL, primary central nervous system lymphoma; DLBCL, diffuse large B-cell lymphoma; FL, follicular lymphoma; nd, not done. The bold value highlights the percentage of B cells, whose relevance is described in the text.

### Immunophenotype of Peripheral Blood Lymphocytes

**Figure 3:**
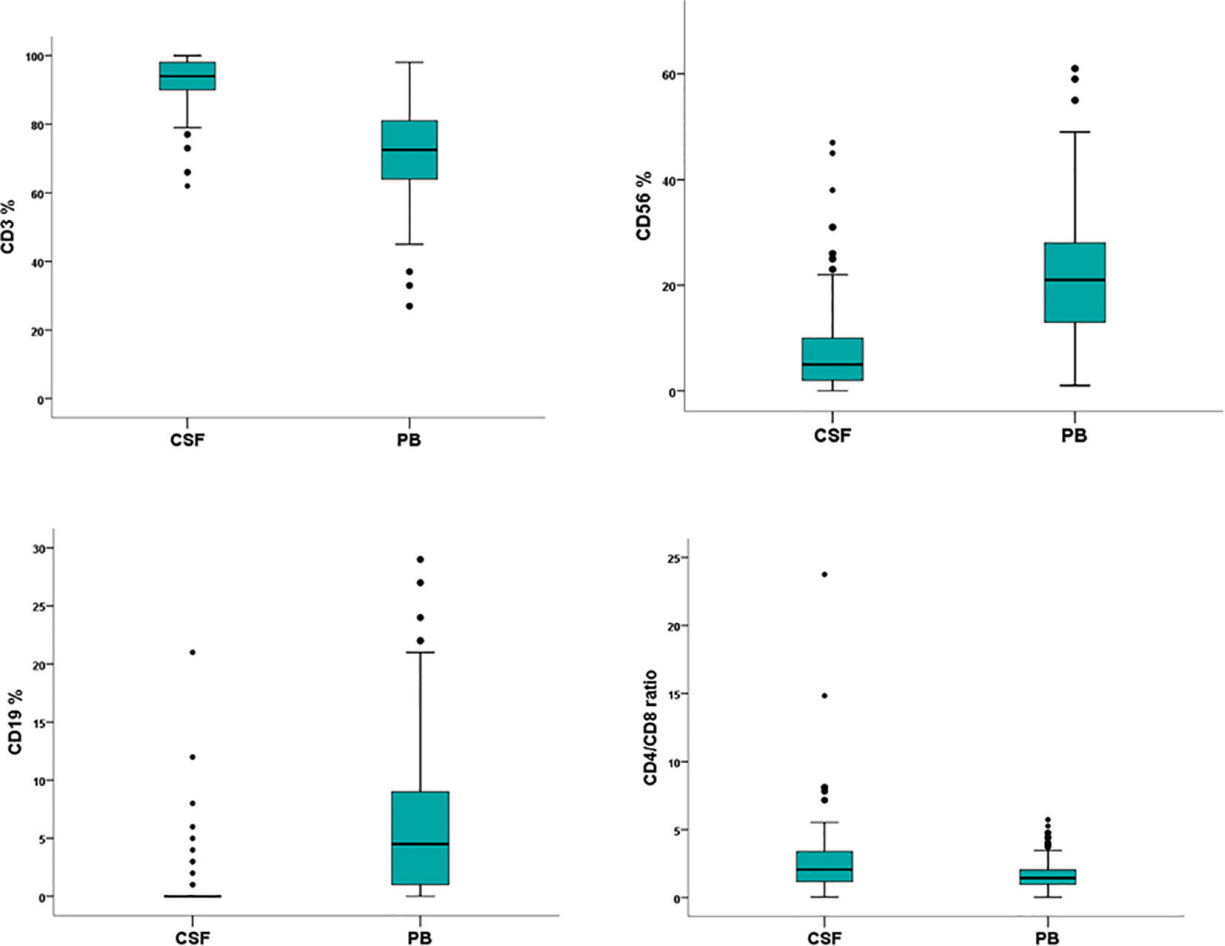
Flow cytometry characterization of cerebrospinal fluid (CSF) and peripheral blood (PB) lymphocytes in 107 non-Hodgkin lymphoma patients negative for leptomeningeal involvement. Wilcoxon rank-sum test documents a significant different distribution between CSF and PB lymphoid subpopulations.

The peripheral blood lymphocyte subset was evaluated in 104 cases (97%) and compared to the corresponding CSF lymphoid subpopulations. The absolute number of lymphocytes was 1100 cell/µl (range 70–3300), the median percentage was 19% (range 2–76). The analysis documented a population of CD3-positive cells (72%, range 27–98) with a CD4/CD8 ratio of 1.4, CD56-positive (21%; range 1–61) and CD19-positive (5%; range 0–29) lymphocytes. A different distribution of CD3 CD56 and CD19 lymphoid subpopulations was observed between CSF and corresponding peripheral blood samples (*p*<0.0001*)* (**Table 2**) (**Figure 3**).

## Discussion

Leptomeningeal metastasis represents one of the greatest challenges in neuro-oncology and CSF is one of the most promising diagnostic tissues utilized in routine clinical practice (13, 24). In addition to cytology, the diagnostic use of flow cytometry is strongly recommended for CSF samples of patients clinically suspected of neoplastic meningitis (12). This report describes the flow cytometry characterization of 138 CSF samples of non-Hodgkin lymphoma patients who underwent diagnostic lumbar puncture according to the routine clinical practice and who were negative for disease infiltration. Knowledge of normal values is essential for diagnostic and research interpretation and, to the best of our knowledge, this is the first, single-institution, report on the flow cytometry characterization of CSF non-neoplastic leukocytes in a large cohort of onco-hematology patients.

The normal cell count of the CSF in adults is up to 4 cells/μL and CSF cell count is a diagnostic criterion for leptomeningeal infiltration. The median cellularity in this cohort of patients was extremely low (1 cell/µl, range 1.0–35), but nevertheless flow cytometry was successfully conducted in 95% of cases, confirming its role of a highly sensitive and specific technique for detection of rare cell sub-populations, even in samples with as few as 1 leukocyte per µl of sample. Of note, in 8% of samples (n=9) the CSF cell count was significantly higher than the normal reference value (median 22 cell/µl). In these cases, unequivocal identification of the cell population was mandatory to exclude false positive interpretation: therefore, in addition to cytology, flow cytometry becomes essential. Moreover, the role of an increased number of lymphocytes in the CSF of non-Hodgkin lymphomas patients deserves to be investigated.

High sensitivity has been reported utilizing a volume of 2.0 ml of CSF for flow cytometry characterization (6). The present study was conducted on a median volume of 4 ml of CSF (≤2 ml being withdrawn in six cases only) with a median of 384 (mean 1518 ± 3772) events analyzed. This number is appreciably higher than the minimum number of events, 10 dots, required for minimal disease identification in CSF flow cytometry (6, 25), confirming the feasibility of comprehensive leukocyte characterization in low volume/low count samples. In this series only 7 cases were not evaluable due to the lack of clustered events. However, cancer cells represent only a proportion, often a minority, of the CSF population in neoplastic meningitis (18, 19, 26). Since there is a positive correlation between the volume of CSF (ml) and the number of events available for flow cytometric analysis (Δ 0.36; *p* < 0.001) and taking into account the potentially extremely low cell count of the CSF, as well as some cell loss related to the staining technique, we recommend the withdrawal of not less than 4 ml of sample to ensure an adequate number of events for a reliable identification of minimally represented sub-populations. Moreover, peripheral blood contamination of the CSF, due to difficulty in the execution of the lumbar puncture, can occur. False positive results represent the major pitfall in all cases with peripheral blood infiltration by leukemic/lymphoma cells and blood contamination of the CSF (author statement manuscript in preparation) (12). Thereafter, discarding the first drops of sample must represent the reference standard in all CSF samples collected for flow cytometry analysis. Moreover, the different distribution of lymphocyte subsets between CSF and peripheral blood can potentially represent a new, useful tool to discriminate between primary and peripheral blood derived populations, particularly at diagnosis or in neutropenic patients.

Studies conducted on normal CSF observed that the vast majority of leucocytes is represented by central memory T lymphocytes with significantly higher percentage compared to blood. The proportion of CD56-positive cells is low while B cells are almost absent (<1%) (27–30). This indicates a selective recruitment of memory T cells into normal CSF. Our study confirms, in a large cohort of samples, that CSF is a tissue rich in CD2 CD3 CD5-positive T lymphocytes with a predominance of CD4-positive over CD8-positive T cells (CD4/CD8 ratio = 2), together with a minority of CD56-positive cells in patients with B-cell non-Hodgkin lymphomas negative for leptomeningeal involvement. The presence of normal T cells in the CSF sample represents not only a strong, reliable internal quality control of the technique but also documents a selective recruitment of T-cell into CSF. The CNS is an immunological sanctuary with restricted access and a unique microenvironment however scientific evidences have recently documented that CNS is no longer an immune-privileged site, but rather a virtual secondary lymphoid organ (31, 32). The tumor inflammatory response is involved in both cancer growth inhibitions as well as in cancer invasiveness (33–37). A relevant proportion of infiltrating T lymphocytes and monocytes beside cancer cells has been documented in patient with breast cancer neoplastic meningitis, with a significant difference in the lymphoid immunophenotype between CSF and peripheral blood (18). Likewise, a sub-population of T cells has been identified in CSF samples positive for B non-Hodgkin lymphoma infiltration (7, 19). Moreover, an active mechanism of reactive CD8 T-lymphocyte migration through the blood-brain barrier has been consistently shown in PCNSL (20, 21). In this study, the ratio between CD4/CD8-positive T cells was shifted significantly in favor of CD4-positive T cells in CSF compared to corresponding peripheral blood (ratio = 2 versus ratio=1.4 respectively; *p *< 0.0001). This different distribution documents that the brain barrier actively selects a sub-population of T lymphocytes, supporting the involvement of the meningeal lymphatic network in lymphoid cell migration into the meninges as a potential alternative route to the cardiovascular system. This finding documents the existence of an active mechanism of lymphocyte localization and provides a promising rationale for the investigation of cellular immunotherapy in brain diseases.

B cells were by far the smallest subset in the CSF of this cohort of B non-Hodgkin lymphoma patients without CNS involvement. The number of B cells is hardly above detection limit in normal CSF (27–29). By contrast, in patients with paraneoplastic neurological syndrome CSF B cell counts showed significantly elevated numbers compared to normal control, suggesting that B lymphocytes are recruited to CSF in certain pathological conditions (38). In the present study, sporadic/no B cells (<10 clustered events) were identified in 90% of the samples and represented a minority (4%) of the lymphoid population in eleven cases (**Table 3**). The normal kappa/lambda *ratio*, evaluated on the CD22-positive population, was crucial for reporting the flow cytometry as negative for infiltration by clonal B cells. A possible correlation between the CSF lymphocyte subpopulations and diagnosis was evaluated. A normal, non-clonal B-cell subpopulation was identified at diagnosis in 71% (5/7) CSF samples of patients with PCNSL (*p* < 0.0001). In contrast with its low frequency in normal CSF and systemic non-Hodgkin lymphomas, the identification of a subpopulation of B cells in the CSF samples of PCNSL cases raises the question of a possible role of blood-brain barrier cell permeability in the pathogenesis of cerebral B-cell lymphomas. Due to the small number of PCNSL cases evaluated, validation on a larger cohort of patients is warranted to confirm this finding and to investigate the role of the CSF B cells in the pathogenesis and diagnosis of the B-cell lymphomas of the brain. A small clonal B‐cell population has been described in the CSF of patients with B‐cell lymphoproliferative disorders and multiple sclerosis suggesting that this finding is not diagnostic of clinically significant involvement of the CNS by lymphoid malignancy (39, 40). Although a larger prospective study with a long follow‐up is required to validate this finding, the identification of a minority of clonal B cells by flow cytometry at diagnosis deserves a careful clinical and instrumental evaluation and more definitive evidence of CNS lymphoid malignancy before a potentially toxic treatment is given (41).

Finally, after (CD4-positive) T lymphocytes, monocytes represent the second most common leukocyte population of the CSF (15%; range 0–70%). Origin and turnover of this medium-size population, well represented in the CSF, is still largely unexplored. The role of monocytes regarding the CNS cellular immune surveillance and their involvement in onco-hematological meningitis deserves in-depth studies and attention.

## Conclusions

The cellular composition of the CSF in non-Hodgkin lymphoma patients negative for leptomeningeal involvement differs profoundly from peripheral blood regarding all major lymphocyte subpopulation. CSF cells are represented by T lymphocytes, in prevalence CD4-positive, and monocytes. B cells are rare and this analysis reveals a possible link with PCNSL. This real-life study confirms the critical role of flow cytometry in routine clinical practice for unequivocal characterization of CSF populations, even in samples with an extremely low cell count. The identification of clusters of normal T cells in the CSF represent a reliable internal quality control of the technique and the significant difference between CSF and paired peripheral blood lymphoid phenotype provides evidence of an independent cerebral lymphatic system. CSF is not an immune-privileged site anymore but a virtual secondary lymphoid organ. An in-depth knowledge of the function and role of the CSF immunological sanctuary is highly needed and has the potential to revolutionize the management of CNS diseases.

## Data Availability Statement

The datasets analyzed for this study can be found in the GARRbox (https://gbox.garr.it/garrbox/index.php/s/6BY66PX6n3LaYZP).

## Ethics Statement

The Central Ethical Committee IRCCS Lazio, Section I.F.O. approved this retrospective study. Protocol no 0009524, July 27, 2020. Written informed consent for participation was not required for this study in accordance with the national legislation and the institutional requirements.

## Author Contributions

IC: designed the study and draft the initial manuscript. SM and APas: flow cytometry studies. IC and SM: analysis, data collection, and quality control. DG: performed the statistical analysis and manuscript revision. MM: histopathological diagnosis and manuscript revision. ST, APac, AM, LC, EP, and PF: patient’s clinical management and critical manuscript revision. All authors contributed to the article and approved the submitted version.

## Conflict of Interest

The authors declare that the research was conducted in the absence of any commercial or financial relationships that could be construed as a potential conflict of interest.
